# Is Exercise Enough? Evidence from Controlled Clinical Trials on Rehabilitation with and Without Adjunct Modalities for Musculoskeletal Disorders

**DOI:** 10.3390/life16040608

**Published:** 2026-04-07

**Authors:** Bindiya Rawat, Yajuvendra Singh Rajpoot, Sohom Saha, Vasile-Cătălin Ciocan, Alina-Mihaela Cristuta, Suchishrava Choudhary, Prashant Kumar Choudhary, Carmina-Mihaela Gorgan, Constantin Sufaru, Nicolae Lucian Voinea

**Affiliations:** 1 Department of Liberal Arts and Social Sciences, Manipal University Jaipur, Jaipur 303007, Rajasthan, India; bindiya.rawat@jaipur.manipal.edu; 2 Department of Sports Management & Coaching, Lakshmibai National Institute of Physical Education, Gwalior 474002, Madhya Pradesh, India; yajupitu25@gmail.com; 3 Department of Sport Psychology, Lakshmibai National Institute of Physical Education, Gwalior 474002, Madhya Pradesh, India; sohomsaha77@gmail.com; 4 Faculty of Movement, Sports, and Health Sciences, ”Vasile Alecsandri” University of Bacau, 600115 Bacau, Romania; cristuta.alina@ub.ro (A.-M.C.); gorgan.carmina@ub.ro (C.-M.G.); lucian.voinea@ub.ro (N.L.V.); 5 Department of Physical Education Pedagogy, Lakshmibai National Institute of Physical Education, Gwalior 474002, Madhya Pradesh, India; suchishrava05@gmail.com (S.C.); prashantlnipe2014@gmail.com (P.K.C.)

**Keywords:** musculoskeletal health, osteoarthritis, tendinopathy, sarcopenia, low back pain, neuromuscular training

## Abstract

Background: Musculoskeletal disorders (MSDs) are a major contributor to global disability. Exercise-based rehabilitation is widely recommended as first-line management; however, in clinical practice, it is frequently combined with adjunct therapeutic modalities, and the incremental effectiveness of these approaches remains unclear. The present review addressed the research question: Do adjunct modalities provide additional benefits beyond exercise-based rehabilitation alone in individuals with musculoskeletal disorders? Methods: This systematic review was conducted according to PRISMA 2020 guidelines and prospectively registered in the PROSPERO database (registration number CRD420261309183). Electronic searches were performed in PubMed/MEDLINE, Scopus, Web of Science, and the Cochrane Central Register of Controlled Trials to identify controlled clinical trials evaluating exercise-based rehabilitation delivered alone or combined with adjunct modalities. Outcomes included pain, functional disability, physical performance, strength, structural or imaging-based measures, biomechanical variables, injury risk, and work-related outcomes. Due to methodological heterogeneity across studies, a structured narrative and tabular synthesis were performed. Results: Twenty-one controlled clinical trials were included, encompassing tendinopathies (*n* = 7), knee osteoarthritis (*n* = 5), post-ACL reconstruction (*n* = 2), chronic spinal pain (*n* = 3), sarcopenia (*n* = 2), low bone mass (*n* = 2), and occupational musculoskeletal conditions (*n* = 1), with sample sizes ranging from 22 to 823 participants. Pain outcomes were reported in 18 studies (86%) and functional outcomes in 16 studies (76%). Exercise-based rehabilitation consistently produced clinically meaningful improvements across studies, whereas adjunct modalities demonstrated short-term advantages in a limited number of trials but rarely showed sustained long-term superiority. Conclusions: Evidence from controlled clinical trials indicates that exercise-based rehabilitation is an effective primary intervention for improving pain, functional capacity, and physical performance across diverse musculoskeletal conditions. Adjunct modalities may provide condition-specific or short-term benefits but do not consistently enhance long-term outcomes beyond structured exercise programs.

## 1. Introduction

Musculoskeletal disorders (MSDs) represent one of the leading causes of pain, physical disability, and reduced quality of life worldwide, imposing a substantial burden on individuals, healthcare systems, and economies [1,2,3]. Conditions such as tendinopathies, osteoarthritis, chronic low back and neck pain, sarcopenia, and post-surgical musculoskeletal impairments are highly prevalent across the lifespan, affecting both clinical and athletic populations. Global estimates indicate a steady rise in the incidence and disability-adjusted life years attributable to MSDs, driven by population aging, sedentary lifestyles, occupational demands, and increased participation in recreational and competitive sports [2,4]. Despite advances in diagnostic imaging and interventional medicine, long-term outcomes for many musculoskeletal conditions remain suboptimal, underscoring the need for effective, sustainable rehabilitation strategies. Exercise-based rehabilitation is widely recognized as the cornerstone of conservative management for MSDs. Across diverse conditions, structured exercise interventions such as resistance training, eccentric loading, neuromuscular training, aerobic conditioning, balance training, and motor control exercises have demonstrated beneficial effects on pain reduction, functional capacity, physical performance, and tissue health [5,6]. Mechanistically, exercise promotes neuromuscular adaptations, improves load tolerance, modulates inflammatory pathways, enhances musculoskeletal metabolism, and supports psychological well-being [7,8]. Exercise-based interventions are increasingly recognized not only for symptom reduction but also for their capacity to modify biomechanical and neuromuscular factors linked to musculoskeletal injury risk. Evidence indicates that changes in neuromuscular coordination, movement variability, and load distribution can influence tissue stress and injury susceptibility, highlighting the value of structured, movement-focused rehabilitation [9,10]. Accordingly, exercise should be positioned as a primary therapeutic strategy rather than an adjunct in musculoskeletal rehabilitation programs.

Consequently, contemporary clinical guidelines consistently recommend exercise as a first-line intervention for most chronic and degenerative musculoskeletal conditions. However, exercise rehabilitation is rarely delivered in isolation within real-world clinical practice. A wide range of adjunct modalities are frequently combined with exercise programs, including extracorporeal shockwave therapy (ESWT), photobiomodulation (PBM), manual therapy, biofeedback, orthoses, digital rehabilitation platforms, and injection-based interventions [11,12]. These adjuncts are proposed to enhance pain modulation, accelerate tissue healing, improve motor learning, or increase adherence to exercise regimens. While some adjunct therapies have demonstrated short-term benefits in specific populations, their incremental value beyond exercise alone remains a matter of debate. Evidence from randomized controlled trials has yielded inconsistent findings regarding the superiority of combined interventions over exercise-only approaches. In Achilles tendinopathy, for example, studies comparing eccentric exercise with ESWT or PBM have reported mixed results, with several trials indicating comparable improvements in pain and function across groups [13,14,15]. Similarly, in knee osteoarthritis, the addition of manual therapy or booster sessions to exercise has shown modest short-term benefits but limited long-term superiority [16,17]. In contrast, certain adjunctive strategies, such as neuromuscular training with real-time biofeedback or high-intensity resistance and impact training for low bone mass, appear to confer condition-specific advantages [18,19]. Beyond clinical efficacy, emerging rehabilitation models increasingly incorporate digital and remotely delivered exercise interventions. Digital care programs and telehealth-based rehabilitation have demonstrated comparable outcomes to traditional in-person physiotherapy for conditions such as chronic low back pain and knee osteoarthritis, while offering potential advantages in accessibility, adherence, and scalability [12,20,21]. These developments are particularly relevant in the context of global disparities in rehabilitation access and healthcare infrastructure, especially in low- and middle-income regions [2].

Despite the growing body of controlled clinical trials, the literature remains fragmented across conditions, intervention types, outcome domains, and methodological designs. Many trials focus on single diagnoses or specific adjunct modalities, limiting the ability to draw overarching conclusions regarding the relative effectiveness of exercise-based rehabilitation with or without adjunct therapies. Furthermore, variations in exercise prescription variables, comparator conditions, follow-up duration, and outcome selection complicate evidence synthesis and clinical translation [7]. Systematic reviews and meta-analyses have typically examined isolated intervention categories such as ESWT, neuromuscular training, or digital exercise rather than adopting an integrative framework that positions exercise as the central therapeutic component [22,23,24].

Although a growing number of controlled clinical trials in musculoskeletal rehabilitation exist, existing evidence remains fragmented by condition, intervention type, and outcome domain, limiting clear conclusions regarding the relative effectiveness of exercise-based rehabilitation with or without adjunct modalities. Previous reviews have predominantly evaluated isolated interventions rather than adopting an integrative, exercise-centered framework. The novelty of the present review lies in positioning exercise as the primary intervention while systematically examining the incremental effects of adjunct therapies across multiple musculoskeletal conditions. Although the review includes a range of musculoskeletal conditions, the analytical framework was intentionally exercise-centered rather than disease-specific. Exercise-based rehabilitation represents a core therapeutic strategy across multiple musculoskeletal disorders despite differences in pathophysiology, and several contemporary rehabilitation frameworks highlight shared mechanistic pathways such as neuromuscular adaptation, load tolerance, and functional restoration. Therefore, examining diverse conditions within a unified framework allows identification of consistent patterns regarding the incremental value of adjunct modalities when exercise is used as the primary therapeutic component. To enhance interpretability, findings were organized and synthesized according to condition-specific outcome domains (e.g., tendinopathy, osteoarthritis, spinal pain, and sarcopenia-related outcomes) and by key rehabilitation targets such as pain, functional disability, structural adaptations, and biomechanical variables.

In this context, the central scientific object of the present study is the effectiveness of exercise-based rehabilitation as the primary therapeutic intervention in musculoskeletal rehabilitation. While numerous studies have investigated specific modalities or isolated treatment strategies, fewer syntheses have examined exercise as the core component of rehabilitation programs while evaluating the incremental contribution of adjunct therapeutic modalities. Clarifying whether exercise alone is sufficient to achieve clinically meaningful outcomes or whether additional modalities provide measurable benefits is essential for improving evidence-based rehabilitation practice.

Accordingly, the primary objective of this systematic review was to compare the effectiveness of exercise-based rehabilitation delivered alone versus exercise combined with adjunct modalities in controlled clinical trials involving individuals with musculoskeletal disorders. Specifically, the review aimed to determine whether adjunct interventions provide meaningful additional benefits beyond structured exercise programs across outcomes related to pain, functional disability, physical performance, structural adaptation, and biomechanical variables. Specifically, outcomes related to pain, functional disability, physical performance, structural adaptations, biomechanical variables, injury risk, and work-related measures were examined to inform evidence-based clinical decision-making and future research.

## 2. Materials and Methods

### 2.1. Eligibility Criteria

The eligibility criteria were predefined to ensure inclusion of studies directly relevant to exercise-based rehabilitation for musculoskeletal disorders, consistent with contemporary evidence identifying exercise as a first-line intervention across a broad range of musculoskeletal conditions [25,26,27]. Studies were selected using a PICOS framework, encompassing human participants aged 9 years and older with clinically diagnosed musculoskeletal, orthopedic, or neuromuscular conditions, including tendinopathies, knee osteoarthritis, post-anterior cruciate ligament reconstruction, chronic neck or low back pain, sarcopenia, low bone mass, and injury-related or occupational musculoskeletal conditions [28,29,30,31]. Eligible interventions were required to include exercise-based rehabilitation as a primary or mandatory component, delivered either alone or in combination with adjunct modalities such as extracorporeal shockwave therapy, photo biomodulation, manual therapy, biofeedback, digital rehabilitation, orthoses, or injection-based treatments, reflecting current multimodal rehabilitation practices [32,33,34,35,36]. Comparators included alternative exercise programs, exercise alone, sham or placebo interventions, usual care, or attention-control conditions. Studies were required to report at least one quantitative outcome related to pain, functional disability, physical performance, strength, biomechanical variables, imaging-based structural outcomes, injury risk, or work-related outcomes, in line with recommended outcome domains for musculoskeletal rehabilitation research [37,38]. Only randomized controlled trials and controlled clinical trials published as full-text, peer-reviewed articles in English were included, following established methodological guidance for evidence synthesis and rehabilitation trials [39,40,41]. Observational studies, case reports, qualitative studies, conference abstracts, animal studies, and trials without a structured exercise component were excluded. The eligibility criteria and outcome domains were prespecified according to the Population–Intervention–Comparator–Outcome (PICO) framework and aligned with the registered PROSPERO protocol. This systematic review was prospectively registered with the International Prospective Register of Systematic Reviews (PROSPERO; registration number CRD420261309183). All eligibility criteria, outcomes, and planned synthesis methods were predefined and are consistent with the registered protocol. The review protocol was prospectively registered in the International Prospective Register of Systematic Reviews (PROSPERO; registration number CRD420261309183). The protocol specified eligibility criteria, outcome domains, and planned synthesis methods prior to data extraction. During the review process, no major deviations from the registered protocol occurred. Minor refinements related to the classification of outcome domains and presentation of narrative synthesis tables were implemented to improve clarity and reporting transparency; however, these changes did not affect the predefined eligibility criteria, study selection process, or overall methodological framework.

The term “exercise-based rehabilitation” encompasses a broad spectrum of structured physical training interventions, including resistance training, eccentric loading, neuromuscular training, motor control exercises, aerobic conditioning, and functional movement programs. To address this variability, studies were included only when exercise constituted a structured, progressive component of the rehabilitation intervention rather than a general physical activity recommendation. Furthermore, exercise interventions were analyzed within outcome domains and clinical contexts to ensure that comparisons remained meaningful despite differences in specific exercise modalities.

### 2.2. Information Sources and Search Strategy

A comprehensive literature search was conducted across multiple electronic databases to identify relevant controlled clinical trials published from January 2000 to December 2025. The databases searched included PubMed/MEDLINE, Scopus, Web of Science, and Cochrane Central Register of Controlled Trials. The search strategy combined controlled vocabulary terms and free-text keywords related to musculoskeletal disorders, exercise-based rehabilitation, and adjunct therapeutic modalities, using Boolean operators to maximize sensitivity. An example search strategy for PubMed included combinations of terms such as musculoskeletal disorders, exercise therapy, resistance training, neuromuscular training, rehabilitation, shockwave therapy, manual therapy, and randomized controlled trial. Reference lists of included studies and relevant reviews were manually screened to identify additional eligible articles.

The complete electronic search strategy for each database, including Boolean operators, truncation terms, and search limits, is provided in Supplementary Materials to ensure transparency and reproducibility in accordance with PRISMA 2020 recommendations. An example of the PubMed search strategy included combinations of the following terms: (“musculoskeletal disorder*” OR “tendinopathy” OR “osteoarthritis” OR “low back pain” OR “sarcopenia”) AND (“exercise therapy” OR “resistance training” OR “neuromuscular training” OR “rehabilitation”) AND (“shockwave therapy” OR “manual therapy” OR “biofeedback” OR “photobiomodulation” OR “digital rehabilitation”) AND (“randomized controlled trial” OR “controlled clinical trial”).

Table 1 presents the predefined inclusion and exclusion criteria structured according to the PICO framework, outlining the eligible population characteristics, intervention and comparator requirements, outcome domains, and study designs.

This table ensured a transparent and consistent study selection process focused on controlled clinical trials evaluating exercise-based rehabilitation for musculoskeletal disorders.

### 2.3. Selection Process

**Figure 1:**
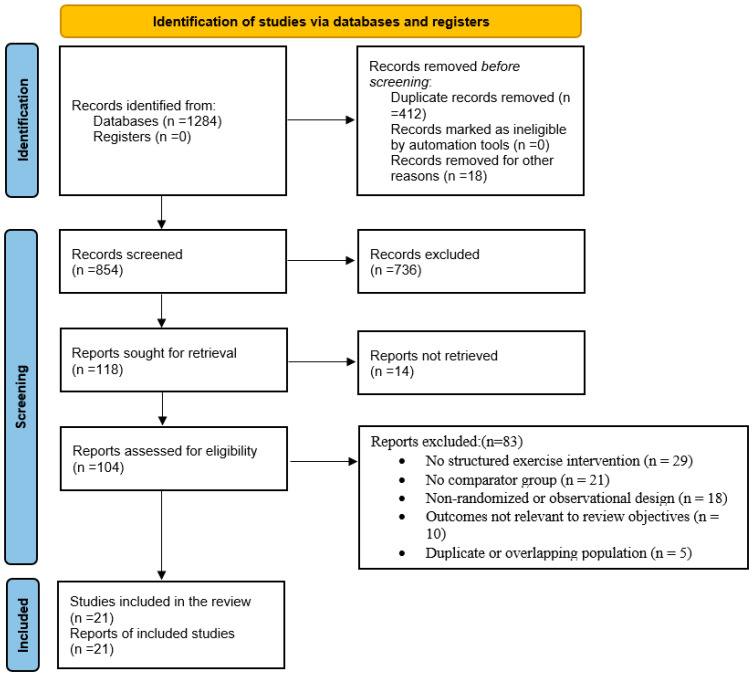
PRISMA 2020 flow diagram of study selection.

Study selection was performed using a two-stage screening process in accordance with established systematic review guidelines. In the first stage, titles and abstracts of all identified records were screened for relevance using predefined eligibility criteria. In the second stage, full-text articles of potentially eligible studies were retrieved and assessed for inclusion. Screening at both stages was conducted independently by two reviewers to minimize selection bias. Any discrepancies or disagreements regarding study eligibility were resolved through discussion and, when necessary, consultation with a third reviewer. The overall selection process and reasons for exclusion at each stage were documented and summarized using a PRISMA 2020 flow diagram (See Figure 1), consistent with current reporting standards for systematic reviews [40,41].

### 2.4. Data Extraction Process

Data extraction was carried out using a standardized and piloted data extraction form to ensure consistency, transparency, and accuracy of collected information, as recommended for evidence synthesis in rehabilitation research [40,42]. Extracted data included study characteristics (author, year, study design), participant characteristics (sample size, age, clinical condition), intervention details (type, duration, intensity, and use of adjunct modalities), comparator conditions, outcome measures, follow-up duration, and key results. Data extraction was performed independently by two reviewers, and all extracted information was cross-checked for accuracy. Any discrepancies were resolved through consensus discussion to ensure data reliability. Where available, quantitative indicators of intervention effects such as mean differences, percentage improvements, or standardized outcome changes reported within the original trials were extracted to assist interpretation of the magnitude and clinical relevance of findings. Because the included studies frequently used different outcome scales and statistical reporting formats, standardized pooled effect estimates were not calculated. Instead, effect magnitude was interpreted descriptively based on reported between-group differences, confidence intervals, minimal clinically important change thresholds, and relative improvements across intervention arms. Where sufficient statistical data were available, standardized effect sizes (standardized mean differences, SMD) were calculated or derived to facilitate comparison of intervention effects across studies with heterogeneous outcome measures. However, in several trials, standardized effect sizes were not extractable for all studies due to incomplete reporting of standard deviations, use of non-parametric analyses, or presentation of outcomes as percentage changes or model-adjusted estimates. In such cases, effect magnitude was interpreted using reported mean differences, confidence intervals, and clinically meaningful thresholds.

### 2.5. Risk of Bias Assessment

**Figure 2:**
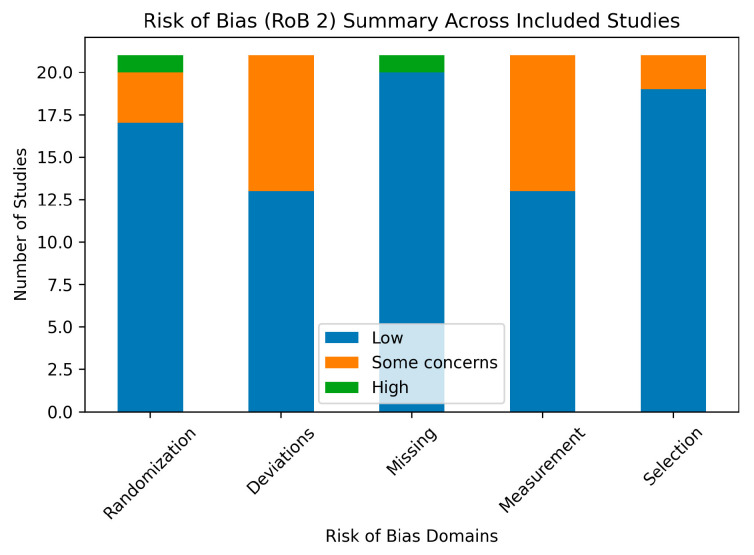
Graphical summary of risk-of-bias assessment across included studies.

The methodological quality and risk of bias of the included studies were assessed using the Cochrane Risk of Bias 2 (RoB 2) tool for randomized controlled trials, which is the recommended instrument for evaluating internal validity in contemporary intervention studies [40]. The RoB 2 tool evaluates bias across five domains: the randomization process, deviations from intended interventions, missing outcome data, measurement of outcomes, and selection of the reported results. Each domain was rated as low risk, some concerns, or high risk of bias, leading to an overall risk-of-bias judgment for each study. Risk of bias assessments were conducted independently by two reviewers, with disagreements resolved through discussion, in line with best-practice recommendations for rehabilitation trials [39]. A graphical summary of the risk-of-bias assessment across studies (RoB 2 summary plot) is presented in Figure 2 to provide a visual overview of methodological quality across domains.

### 2.6. Data Synthesis Methods

Given the substantial heterogeneity across included studies in terms of participant populations, musculoskeletal conditions, intervention characteristics, comparator groups, outcome measures, and follow-up durations, a quantitative meta-analysis was not considered appropriate. In addition to clinical heterogeneity, methodological heterogeneity across studies was evaluated during the synthesis planning stage. Key sources of variability included differences in exercise prescription variables (intensity, frequency, supervision), adjunct modality types, outcome measurement instruments, and follow-up durations. Preliminary evaluation indicated that the available trials did not provide sufficiently comparable outcome metrics to calculate pooled effect estimates using standard statistical measures such as the I^2^ statistic or Cochran’s Q test. Because many studies used condition-specific outcome scales and heterogeneous comparator designs, statistical pooling would have produced unreliable or potentially misleading summary estimates. Therefore, consistent with methodological recommendations for complex rehabilitation interventions, a structured narrative synthesis was considered more appropriate.

Before adopting a narrative synthesis approach, the feasibility of quantitative meta-analysis was explored for subsets of studies with apparently similar clinical conditions. However, pooling was not considered methodologically appropriate due to substantial heterogeneity in several critical dimensions, including intervention characteristics (exercise prescription variables, adjunct modality type, dosage, and supervision), comparator conditions, follow-up durations, and outcome measures. Even within condition-specific subsets such as Achilles tendinopathy or knee osteoarthritis, trials frequently employed different outcome instruments, intervention intensities, or co-interventions, which would have introduced substantial statistical and clinical heterogeneity. Consistent with current methodological guidance for complex rehabilitation interventions, a structured narrative synthesis was therefore adopted to allow transparent comparison of intervention effects across studies while preserving clinical interpretability.

Consequently, a narrative and tabular synthesis approach was adopted, consistent with guidance for synthesizing complex and heterogeneous rehabilitation interventions [40]. Study findings were synthesized descriptively and organized into predefined outcome domains, including pain, functional disability, physical performance and strength, structural and imaging-based outcomes, biomechanical variables, injury risk, and work-related outcomes, which reflect recommended outcome frameworks in musculoskeletal rehabilitation research [37,38,43]. Summary tables were used to present the direction and consistency of effects across studies, facilitating comparison between exercise-only interventions and exercise combined with adjunct modalities. The feasibility of subgroup meta-analysis was further explored for clinically comparable subsets of studies, particularly within tendinopathy and knee osteoarthritis populations. However, quantitative pooling was not considered appropriate due to persistent heterogeneity in intervention protocols, including variations in exercise intensity, frequency, supervision, and adjunct modality application. Additionally, inconsistencies in outcome measurement instruments and statistical reporting formats across studies limited direct comparability. As a result, conducting a meta-analysis, even at the subgroup level, was deemed methodologically inappropriate, and a structured narrative synthesis was maintained to ensure both analytical rigor and clinical relevance.

## 3. Results

The results of this review are based on 21 controlled clinical trials evaluating exercise-based rehabilitation delivered alone or in combination with adjunct modalities across a broad range of musculoskeletal conditions. Although the included trials covered several musculoskeletal conditions, the distribution of studies across conditions was not uniform. Tendinopathies and knee osteoarthritis accounted for a substantial proportion of trials, reflecting the strong evidence base and high clinical research activity within these conditions. Other conditions, such as sarcopenia, low bone mass, and occupational musculoskeletal disorders, were represented by fewer controlled trials meeting the eligibility criteria. Therefore, the review provides a broad but uneven representation of the current evidence base, which reflects the existing distribution of controlled rehabilitation trials rather than a predetermined emphasis on specific diagnoses.

The included studies encompassed diverse clinical and community populations and investigated outcomes related to pain, functional disability, physical performance, strength, structural and imaging-based adaptations, biomechanical variables, injury risk, and work-related outcomes. Overall, the findings provide a comprehensive overview of the direction and consistency of effects associated with exercise-centered rehabilitation interventions, while highlighting variability in intervention approaches, comparator conditions, and outcome domains across studies.

In interpreting the findings, particular emphasis was placed on between-group comparisons reported within the controlled trials, as these comparisons provide the most relevant evidence regarding the incremental effectiveness of adjunct modalities relative to exercise-only interventions. While within-group improvements were reported in many studies, the synthesis prioritized differences between intervention arms and the magnitude of comparative effects where available, in order to more directly address the central research question of whether adjunct modalities enhance outcomes beyond exercise-based rehabilitation alone.

Where available, key quantitative indicators reported within the original trials (including *p*-values, mean changes, or clinically important difference thresholds) were summarized to facilitate interpretation of intervention effects. However, due to variation in outcome reporting formats and measurement scales across studies, standardized effect size metrics were not consistently extractable. Therefore, results are presented descriptively while highlighting the magnitude and direction of between-group differences reported in each trial.

Table 2 summarizes the characteristics of 21 controlled clinical trials evaluating exercise-based rehabilitation across diverse musculoskeletal conditions, including tendinopathies, knee osteoarthritis, post-ACL reconstruction, chronic spinal pain, sarcopenia, low bone mass, and occupational health populations. Sample sizes ranged from small laboratory-based trials to large multicentre studies involving more than 800 participants.

Interventions varied widely and included eccentric loading, resistance and neuromuscular training, high-intensity resistance and impact training, and home-based or digital exercise programs. Several trials examined exercise alone, while others assessed exercise combined with adjunct modalities such as shockwave therapy, photo biomodulation, manual therapy, biofeedback, dietary intervention, or injections. Outcomes commonly assessed were pain, functional disability, physical performance, strength, structural or imaging measures, and biomechanical variables, with follow-up periods ranging from short-term to two years.

Table 3 presents the risk of bias assessment for the included studies using domain-based evaluation criteria. Most trials demonstrated a low risk of bias in the randomization process and outcome measurement domains, reflecting adequate random sequence generation and use of validated outcome measures. Some concerns were identified in several studies regarding deviations from intended interventions and selective reporting, primarily due to the limited blinding inherent to exercise-based interventions and insufficient detail regarding adherence monitoring.

Missing outcome data posed a high risk of bias in a small number of studies, particularly where dropout rates were substantial or incompletely reported. One semi-randomized trial was judged to have an overall high risk of bias due to limitations in allocation procedures. Overall, the majority of included trials were rated as low risk or presented some concerns, indicating generally acceptable methodological quality across the evidence base.

To facilitate comparison across studies, the summary tables highlight the intervention, comparator group, primary outcome domain, and the main between-group findings reported in each trial.

Table 4 summarizes pain-related outcomes across studies evaluating exercise-based rehabilitation with or without adjunct modalities. Pain outcomes were most frequently measured using validated scales such as the visual analog scale, VISA-A, and WOMAC pain subscale. Across tendinopathy, osteoarthritis, neck pain, and post-surgical populations, exercise interventions consistently resulted in significant pain reductions from baseline.

In several trials, adjunct modalities such as photo biomodulation, manual therapy, or shockwave therapy provided short-term or condition-specific advantages; however, between-group differences were often modest or time-dependent. In Achilles tendinopathy, eccentric exercise demonstrated comparable or superior long-term pain reduction compared with adjunct therapies. In knee osteoarthritis and chronic neck pain, targeted exercise approaches produced meaningful improvements in pain outcomes, with neuromuscular and motor control-based interventions frequently outperforming conventional strengthening programs.

Outcome reporting was standardized across tables by presenting the primary functional measure used in each study together with the principal comparative finding between intervention groups.

Table 5 presents functional and disability-related outcomes reported across the included studies. Functional status was assessed using condition-specific instruments such as the Shoulder Pain and Disability Index, DASH, WOMAC, Oswestry Disability Index, and Neck Disability Index. Most exercise-based interventions led to clinically meaningful improvements in functional outcomes regardless of musculoskeletal condition.

Comparisons between different exercise modalities often revealed similar functional gains, while certain adjunct interventions, such as injection-based treatments or structured manual therapy programs, demonstrated short-term superiority in selected populations. Digital and home-based exercise programs produced functional improvements comparable to traditional in-person physiotherapy, with additional benefits related to adherence and feasibility. Overall, the findings indicate that exercise-based rehabilitation is effective in improving functional capacity across diverse musculoskeletal disorders.

Table 6 integrates outcomes related to physical performance, musculoskeletal structure, biomechanics, injury risk, and occupational productivity. Strength and performance outcomes, including maximal voluntary contraction, walking capacity, and functional performance tests, consistently improved following resistance, neuromuscular, and high-intensity training interventions across clinical and community-based populations.

Structural and imaging outcomes demonstrated that exercise, particularly high-intensity resistance and impact training, was associated with favorable adaptations in bone mineral density, cortical thickness, and tendon structure, while adjunct modalities showed condition-specific effects on tissue properties. Biomechanical analyses revealed that improvements in symptoms and strength were not always accompanied by changes in joint loading patterns during functional tasks. Injury-risk outcomes indicated that neuromuscular training combined with targeted biofeedback was effective in modifying biomechanical risk factors in athletic populations. In occupational settings, exercise-based interventions led to reductions in presenteeism and productivity loss compared with non-exercise approaches.

In Table 7, the synthesized evidence demonstrates that exercise-based interventions consistently produce significant improvements in clinical outcomes such as pain, function, strength, and quality of life across diverse musculoskeletal conditions. The addition of adjunct modalities (e.g., ESWT, biofeedback, manual therapy, or biologics) shows condition-specific benefits, with some interventions yielding large effect sizes (e.g., laser therapy, HiRIT, PRP/BMC), while others provide minimal or no additional advantage over exercise alone.

Importantly, several studies reveal a dissociation between clinical improvements and biomechanical adaptations, indicating that symptom relief may not always correspond to structural or loading changes. The magnitude of effects varies widely, ranging from trivial to very large, highlighting heterogeneity in intervention efficacy and outcome responsiveness. Subgroup trends suggest that targeted, high-intensity, or progressive exercise approaches tend to produce superior and sustained outcomes. Overall, the findings support exercise as the core therapeutic strategy, with adjuncts acting as context-dependent performance modulators rather than universally superior treatments. Across the included studies, effect sizes ranged from trivial (SMD ≈ −0.14) to very large (SMD ≈ 2.5), with the majority of interventions demonstrating moderate-to-large effects (SMD ≥ 0.5). Exercise-based interventions consistently produced clinically meaningful improvements across outcome domains, whereas adjunct modalities demonstrated variable effects. Large effect sizes were observed in specific contexts, such as photobiomodulation combined with eccentric exercise and high-intensity resistance training for bone health, while several adjunct interventions showed minimal or no additional benefit beyond exercise alone.

### Condition-Specific Synthesis of Findings

The findings were further synthesized according to major clinical condition categories to enhance interpretability and address clinical heterogeneity across studies.

In studies involving tendinopathies (*n* = 7), exercise-based interventions, particularly eccentric loading protocols, consistently demonstrated significant improvements in pain and function. Adjunct modalities such as extracorporeal shockwave therapy and photobiomodulation showed additional short-term benefits in some trials; however, these effects were not consistently superior to exercise alone at longer follow-up periods. For knee osteoarthritis (*n* = 5), structured exercise interventions resulted in clinically meaningful reductions in pain and improvements in functional outcomes. The addition of manual therapy or booster sessions provided modest short-term benefits, but these advantages were generally not sustained at long-term follow-up, indicating limited incremental value beyond exercise. In post-anterior cruciate ligament reconstruction populations (*n* = 2), neuromuscular training demonstrated superior outcomes compared with traditional strength training with respect to pain reduction, functional performance, and quality of life, underscoring the importance of movement-specific rehabilitation strategies. Among studies examining chronic spinal pain (*n* = 3), including chronic low back pain and neck pain, both conventional and digital exercise-based interventions produced comparable improvements in disability and pain outcomes. Targeted motor control approaches, such as craniocervical flexion exercises, showed superior effects in specific functional parameters. In sarcopenia (*n* = 2), resistance-based exercise interventions consistently improved muscle strength, functional performance, and physical capacity, with no evidence suggesting the necessity of adjunct modalities to achieve clinically meaningful outcomes. For low bone mass and osteoporosis (*n* = 2), high-intensity resistance and impact training demonstrated clear superiority over low-intensity or usual activity interventions in improving bone mineral density and structural outcomes, representing one of the most consistent condition-specific benefits observed. Overall, these subgroup findings indicate that while exercise-based rehabilitation is effective across all conditions, the incremental benefits of adjunct modalities are condition-dependent and are most evident in specific clinical contexts rather than universally applicable.

## 4. Discussion

The present review synthesized evidence from 21 controlled clinical trials evaluating exercise-based rehabilitation delivered either alone or in combination with adjunct modalities across a wide spectrum of musculoskeletal disorders. The primary objective was to position exercise as the central therapeutic component while systematically examining whether adjunct interventions meaningfully enhance rehabilitation outcomes across pain, functional disability, physical performance, structural adaptations, biomechanical variables, injury risk, and work-related outcomes. Overall, the findings indicate that exercise-based rehabilitation consistently produces clinically meaningful improvements across most outcome domains, whereas adjunct modalities tend to provide condition-specific, short-term, or context-dependent benefits rather than robust additive effects.

### 4.1. Exercise as the Foundational Intervention in Musculoskeletal Rehabilitation

Across tendinopathies, knee osteoarthritis, chronic spinal pain, sarcopenia, low bone mass, and post-surgical populations, exercise-based rehabilitation demonstrated consistent improvements in pain, function, and physical performance. These findings reinforce long-standing evidence that exercise constitutes a first-line intervention for musculoskeletal disorders [6,25,26]. Importantly, the present synthesis extends prior work by integrating evidence across traditionally siloed clinical conditions and intervention models, thereby highlighting the generalizability of exercise-centered rehabilitation effects.

Pain outcomes were among the most consistently improved domains. Across Achilles tendinopathy, lateral elbow tendinopathy, knee osteoarthritis, chronic neck pain, and post-ACL reconstruction populations, exercise interventions resulted in significant reductions in pain scores measured using validated tools such as VAS, VISA-A, and WOMAC. These findings align with contemporary umbrella reviews demonstrating that appropriately dosed exercise reduces pain irrespective of musculoskeletal diagnosis, albeit with variability influenced by prescription variables such as intensity, frequency, and progression [7].

Functional and disability-related outcomes similarly improved across conditions. Most trials reported clinically meaningful gains in SPADI, DASH, WOMAC, ODI, and condition-specific functional scales, regardless of whether exercise was delivered in isolation or combined with adjunct therapies. Notably, comparisons between different exercise modalities frequently revealed comparable functional improvements, suggesting that multiple exercise approaches may be effective provided that programs are adequately progressed and individualized [56]. These findings support the growing emphasis on adherence, load management, and behavioral engagement rather than rigid adherence to a single exercise paradigm.

### 4.2. Added Value of Adjunct Modalities: Modest and Condition-Specific Effects

A central contribution of this review lies in its systematic examination of adjunct modalities added to exercise-based rehabilitation. Across included trials, adjuncts such as extracorporeal shockwave therapy, photobiomodulation, manual therapy, biofeedback, dietary intervention, and injection-based treatments demonstrated heterogeneous effects.

In tendinopathy, several trials reported short-term advantages of adjunct therapies. Photobiomodulation combined with eccentric exercise resulted in greater improvements in VISA-A scores compared with placebo laser in the short term [15], while ESWT demonstrated modest time-dependent benefits in pain and function [45]. However, long-term follow-up data frequently favored exercise alone. For example, eccentric exercise maintained pain reduction at two years in Achilles tendinopathy, whereas ESWT effects were less durable [13]. These findings are consistent with recent systematic reviews indicating that while adjunct modalities may accelerate early symptom relief, exercise remains the primary driver of sustained adaptation in tendon structure and function [11,57].

Manual therapy and booster sessions provided short-term benefits in knee osteoarthritis but failed to demonstrate long-term superiority when combined with exercise [16,17]. This pattern aligns with emerging critiques that certain manual therapy trial designs may overestimate clinical effectiveness when longer-term outcomes are considered [35]. Similarly, injection-based interventions demonstrated short-term superiority in selected populations, such as supraspinatus tears treated with bone marrow concentrate and platelet products; however, exercise-only groups continued to improve over time, underscoring the necessity of structured rehabilitation even when regenerative approaches are employed [50].

### 4.3. Strength, Performance, and Structural Adaptations

Exercise-based interventions consistently improved strength and physical performance across sarcopenic, osteoarthritic, and post-surgical populations. Resistance training, neuromuscular training, and high-intensity resistance and impact training produced large gains in muscle strength, functional capacity, and walking performance [43,46,49]. These findings corroborate recent consensus statements identifying resistance-based exercise as a cornerstone intervention for sarcopenia and age-related functional decline [30].

Structural and imaging outcomes further support the role of exercise as a disease-modifying intervention. High-intensity resistance and impact training resulted in improvements in bone mineral density and cortical thickness in both men and women with low bone mass [19,54]. In contrast, adjunct modalities exhibited more selective structural effects, such as changes in tendon elasticity measured via shear wave elastography following point-focused ESWT [14]. These findings suggest that while adjunct therapies may influence specific tissue properties, exercise-induced mechanical loading remains essential for broad musculoskeletal adaptation.

#### Physiological Mechanisms Underlying Exercise-Based Rehabilitation

The beneficial effects of exercise-based rehabilitation in musculoskeletal disorders can be partly explained by several physiological mechanisms. Mechanical loading stimulates adaptive responses in muscle, tendon, bone, and connective tissue through mechanotransduction pathways that regulate collagen synthesis, muscle hypertrophy, and bone remodeling. Resistance and neuromuscular training increase motor unit recruitment, enhance neuromuscular coordination, and improve joint stability, which contributes to reductions in pain and functional limitations. Exercise also influences inflammatory and metabolic pathways by reducing pro-inflammatory cytokines and improving mitochondrial function and local tissue perfusion. Furthermore, regular exercise has been shown to induce central and peripheral analgesic effects through modulation of endogenous opioid systems and improved pain processing. These mechanisms collectively support the role of exercise as a biologically plausible and clinically effective intervention for musculoskeletal rehabilitation [5,6].

### 4.4. Biomechanical Outcomes and Injury-Risk Modification

Biomechanical outcomes demonstrated more variable responses to intervention. Improvements in strength and symptoms were not always accompanied by changes in joint loading patterns during functional tasks, as observed in knee osteoarthritis following quadriceps strengthening (DeVita et al., 2018) [55]. This dissociation highlights the complexity of translating strength gains into biomechanical reorganization. The observed functional and performance-related improvements align with mechanistic evidence demonstrating that targeted exercise interventions can enhance neuromuscular symmetry, trunk-pelvic control, and movement efficiency. Prior biomechanical investigations have shown that neuromuscular asymmetry and impaired lumbopelvic control are associated with elevated injury risk, and that corrective training strategies may mitigate these deficits [58]. Similarly, fatigue-related biomechanical adaptations reported in experimental studies suggest that exercise interventions emphasizing neuromuscular coordination and load management may have important implications for long-term injury prevention and movement sustainability [10].

In contrast, neuromuscular training combined with real-time biofeedback demonstrated favorable effects on biomechanical injury-risk proxies, particularly knee abduction moment in adolescent female athletes [18]. These findings align with broader evidence supporting neuromuscular training for injury prevention, particularly when programs target movement quality and motor control [23,59]. However, the context-specific nature of these effects underscores the need for targeted application rather than routine inclusion of biofeedback across all rehabilitation settings.

### 4.5. Digital and Home-Based Exercise Delivery

Several trials evaluated digital or home-based exercise programs, with outcomes comparable to traditional in-person physiotherapy for chronic low back pain and knee osteoarthritis [20,21]. Digital interventions were associated with lower dropout rates and improved feasibility, supporting their potential role in expanding access to rehabilitation services. These findings are consistent with recent systematic reviews demonstrating that digital exercise interventions can achieve clinically meaningful outcomes when adherence and program progression are adequately supported [12,60].

### 4.6. Methodological Considerations and Quality of Evidence

Risk-of-bias assessment revealed generally acceptable methodological quality across included trials, with most studies rated as low risk or presenting some concerns. Common limitations included incomplete blinding, deviations from intended interventions, and insufficient reporting of adherence, which are inherent challenges in exercise-based trials [39,42]. One semi-randomized trial was judged to have a high overall risk of bias, highlighting the importance of rigorous allocation procedures in future research.

Heterogeneity across populations, interventions, and outcome measures precluded quantitative meta-analysis. However, the narrative and tabular synthesis adopted in this review enabled identification of consistent directional trends and highlighted areas where adjunct therapies may offer incremental benefit. This integrative approach addresses a key gap in the literature, as prior systematic reviews have largely examined isolated intervention categories rather than positioning exercise as the central therapeutic framework [22,24]. Risk-of-bias assessment exhibited limitations related to adherence monitoring, incomplete blinding, or deviations from intended interventions, which may influence the reliability of reported intervention effects. Consequently, conclusions regarding the incremental benefits of adjunct modalities should be interpreted with greater confidence when supported by trials demonstrating low risk of bias. In contrast, findings derived from studies with methodological concerns should be viewed as exploratory and requiring confirmation in rigorously designed randomized trials.

Another consideration relates to the heterogeneity of exercise-based interventions included in this review. Exercise rehabilitation can range from low-intensity mobility programs to high-intensity resistance and neuromuscular training protocols. Although this diversity reflects real-world rehabilitation practice, it may limit direct comparability between trials. Nevertheless, the consistent pattern of clinically meaningful improvements across diverse exercise modalities reinforces the concept that structured mechanical loading and progressive training stimuli represent key therapeutic drivers in musculoskeletal rehabilitation, irrespective of the specific exercise modality employed. A key limitation of this review is the substantial clinical heterogeneity across included studies, encompassing diverse musculoskeletal conditions, intervention protocols, and outcome measures. Although an exercise-centered analytical framework was adopted, the findings should be interpreted as a high-level synthesis rather than condition-specific clinical recommendations. The variability in study designs and reporting formats also limited the ability to derive standardized effect sizes for all trials and precluded quantitative meta-analysis.

### 4.7. Clinical Implications and Future Research Directions

From a clinical perspective, the findings reinforce exercise-based rehabilitation as the cornerstone of musculoskeletal care. Adjunct modalities should be considered selectively, guided by condition-specific evidence, patient preference, and short-term symptom management goals rather than assumed additive efficacy. Clinicians should prioritize optimization of exercise prescription variables, adherence strategies, and long-term progression, which appear to exert greater influence on outcomes than the routine addition of passive or technological adjuncts.

Future research should focus on head-to-head comparisons of exercise-only versus exercise-plus-adjunct interventions with adequate follow-up durations, transparent reporting of adherence, and integration of patient-centered outcomes. Additionally, mechanistic studies linking changes in strength, structure, and biomechanics to clinical outcomes may help clarify why adjunct benefits are often transient or context-specific.

### 4.8. Implications for Healthcare Systems and Rehabilitation Policy

The findings of this review also have implications for health policy and rehabilitation service delivery. Exercise-based rehabilitation programs are generally low-cost, scalable, and accessible interventions that can be implemented in community, clinical, and digital health settings. Given the high global burden of musculoskeletal disorders and the limited availability of specialized rehabilitation services in many regions, prioritizing exercise-centered rehabilitation strategies may improve accessibility and cost-effectiveness of care. Policymakers and healthcare systems should therefore consider integrating structured exercise programs into primary care and community health initiatives while promoting training of healthcare professionals in evidence-based exercise prescription. Such strategies may contribute to reducing healthcare expenditures associated with chronic musculoskeletal conditions while improving long-term functional outcomes for affected populations.

## 5. Conclusions

This review confirms that exercise-based rehabilitation is an effective and reliable primary intervention for improving pain, functional disability, physical performance, and strength across a broad range of musculoskeletal disorders. Consistent benefits were observed in tendinopathies, knee osteoarthritis, post-anterior cruciate ligament reconstruction, chronic spinal pain, sarcopenia, low bone mass, and work-related musculoskeletal conditions. The overall evidence indicates that structured, progressive exercise alone is sufficient to achieve clinically meaningful rehabilitation outcomes in most populations. The addition of adjunct modalities, including extracorporeal shockwave therapy, photobiomodulation, manual therapy, biofeedback, digital platforms, or injection-based treatments, did not consistently enhance long-term outcomes beyond those achieved with exercise-based rehabilitation. When benefits were observed, they were typically short-term, condition-specific, or limited to selected outcome domains. These findings suggest that adjunct therapies should not be applied routinely but rather used selectively, based on individual patient needs, symptom severity, and short-term rehabilitation goals. From a clinical perspective, rehabilitation programs should prioritize optimization of exercise prescription variables, including load progression, intensity, frequency, supervision, and adherence strategies, as these factors appear to exert a greater influence on outcomes than the addition of passive or technology-driven adjuncts. Resistance training, neuromuscular training, and high-intensity loading approaches are particularly recommended where strength, functional performance, bone health, or injury-risk modification are primary targets. Future research should focus on high-quality comparative trials with adequate follow-up durations to determine when adjunct modalities provide meaningful incremental value over exercise alone. Improved reporting of adherence, intervention fidelity, and clinically relevant outcome thresholds is also recommended. Overall, adopting an exercise-centered, evidence-informed rehabilitation framework may enhance clinical efficiency, reduce unnecessary treatment complexity, and support more consistent and durable outcomes in musculoskeletal care. However, these findings should be interpreted within the context of heterogeneity across conditions and interventions.

## Figures and Tables

**Table 1:** Inclusion and exclusion criteria.

PICO Component	Inclusion Criteria	Exclusion Criteria
Population (P)	Human participants aged ≥9 years Diagnosed musculoskeletal, orthopedic, or neuromuscular conditions, including tendinopathies (Achilles, rotator cuff, lateral elbow), knee osteoarthritis, post-ACL reconstruction, chronic neck or low back pain, sarcopenia, osteopenia/osteoporosis, or athletic injury-risk populations Diagnosis established using clinical criteria, imaging, or validated guidelines	Healthy participants without a clinical or rehabilitation purpose Pediatric populations < 9 years Animal or in vitro studies Studies focusing exclusively on neurological, cardiovascular, or systemic diseases
Intervention (I)	Exercise-based rehabilitation as a primary or mandatory component, including resistance training, eccentric exercise, neuromuscular training, aerobic exercise, motor control, balance, or functional training Exercise delivered alone or combined with adjunct modalities (e.g., ESWT, photo biomodulation, manual therapy, biofeedback, digital rehabilitation, orthoses, injections)	Interventions without an exercise component Purely pharmacological, surgical, or injection-only studies without structured rehabilitation Passive treatments used in isolation
Comparison (C)	Active exercise comparator Exercise plus adjunct vs. exercise alone Sham/placebo intervention Usual care or attention control	Studies without a comparator group Single-group pre–post studies
Outcomes (O)	At least one quantitative outcome, including pain (VAS, NPRS), function/disability (SPADI, WOMAC, VISA-A, DASH, ODI), physical performance or strength, biomechanical variables, imaging-based structural outcomes, or injury incidence	Studies reporting only qualitative outcomes Biomechanical or laboratory studies without clinical or functional outcomes
Study Design (S)	Randomized controlled trials, cluster-RCTs, factorial RCTs, crossover RCTs, or semi-randomized controlled trials Peer-reviewed full-text articles in English	Observational studies, case reports, case series

**Table 2:** Characteristics of studies (*n* = 21).

Author & Year	Aim	Population	Intervention	Comparison	Outcome	Study Design	Test Results
Heron et al., 2017 [44]	To compare the efficacy of three rotator cuff loading exercise programs for rotator cuff tendinopathy/shoulder impingement syndrome.	120 adults with shoulder pain for ≥3 months, full passive ROM, pain on rotator cuff tests; NHS physiotherapy departments.	Three exercise programs (6 weeks): open-chain resisted-band, closed-chain loading, or minimally loaded ROM; all with stretching.	Open-chain vs. closed-chain vs. ROM programs.	Primary: SPADI change at 6 weeks. Secondary: MCIC (≥10 points), deterioration, dropout.	Parallel-group RCT (intention-to-treat).	All groups showed significant SPADI reduction (*p* ≤ 0.0002) with no between-group differences. MCIC similar (40–43%); ROM deterioration 10%. Dropout was highest in closed-chain (48%); among completers, closed-chain showed the highest MCIC (76%).
Vahdatpour et al., 2018 [45]	To evaluate ESWT on pain and function in chronic Achilles tendinopathy.	43 adults (18–70 yrs) with chronic Achilles tendinopathy.	ESWT: 4 weekly sessions + conservative care.	Sham ESWT + same care.	Pain (VAS), function (AOFAS).	Double-blind RCT.	Both groups improved (*p* < 0.001). Between-group differences only at 16 weeks (VAS *p* = 0.047; AOFAS *p* = 0.013). No adverse events.
Gatz et al., 2021 [14]	To compare line- vs. point-focused ESWT vs. placebo in Achilles tendinopathy.	66 adults with insertional or midportion Achilles tendinopathy.	Physiotherapy + line- or point-focused ESWT.	Physiotherapy + placebo ESWT.	Primary: VISA-A. Secondary: AOFAS, recovery, tendon structure.	Single-blind placebo-controlled RCT.	VISA-A improved in all groups (*p* < 0.001); no between-group differences. SWE elasticity increased only in point-focused ESWT (*p* ≤ 0.03).
Seo et al., 2021 [46]	To examine resistance training effects in sarcopenic older women.	22 sarcopenic women (>65 yrs).	Bodyweight + elastic-band RT, 16 weeks.	Non-exercise control.	Functional fitness, muscle quality, growth factors.	RCT (MANOVA/ANOVA).	RT improved strength, fitness, MVIC, and RMVIC (*p* < 0.001); prevented IMAT increase; and follistatin ↑ (*p* = 0.038).
Pereira et al., 2019 [47]	To compare ergonomics + neck exercise vs. health promotion on productivity.	763 office workers from 14 organizations.	Ergonomics + neck-specific exercise (12 weeks).	Ergonomics + health promotion.	Productivity loss (HPQ).	Cluster-RCT (1 year).	At 12 months, lower monetised productivity loss and presenteeism in the exercise group (*p* < 0.05).
Messier et al., 2022 [48]	To assess diet + exercise on knee pain in OA.	823 adults ≥ 50 yrs with knee OA and BMI ≥ 27.	Diet + supervised exercise (18 months).	Attention control.	WOMAC pain (primary).	Assessor-blinded RCT.	WOMAC pain was lower in the intervention (*p* = 0.02, below MCID); weight loss and function improved.
Khalid et al., 2022 [49]	To compare neuromuscular vs. strength training post-ACL reconstruction.	76 adults, 2 months post-ACL surgery.	Neuromuscular training (6 weeks).	Strength training.	Pain, function, QoL, strength, power.	Single-blind RCT.	NMT superior for pain, function, QoL, strength, and power (*p* ≤ 0.002).
Cui et al., 2023 [20]	To compare digital vs. in-person physiotherapy for CLBP.	140 adults with CLBP.	Digital care program (8 weeks).	In-person physiotherapy.	ODI (primary).	Parallel-group RCT.	Both groups improved; no between-group differences. The digital group had lower dropout (*p* = 0.019).
Demir Benli et al., 2022 [13]	To compare EE vs. ESWT in Achilles tendinopathy.	63 patients with midportion Achilles tendinopathy.	EE (Alfredson protocol).	ESWT.	Pain, function, tendon structure.	RCT with 2-year follow-up.	Both improved short-term; only EE maintained pain reduction at 2 years.
Centeno et al., 2024 [50]	To compare BMC injection vs. exercise for supraspinatus tears.	51 adults with ≥50% supraspinatus tears.	BMC + PRP + platelet lysate injection.	Exercise therapy.	DASH, pain, SANE, MRI healing.	RCT with crossover.	BMC superior at 3 months; MRI healing in 73%; improvements persisted to 24 months.
Ford et al., 2025 [18]	To examine NMT with biofeedback on KAM.	150 adolescent female soccer players.	NMT + sham/knee/hip biofeedback.	Active comparator arms.	KAM during DVJ and CUT.	Randomized active-comparator trial.	DVJ KAM ↓ in all groups; CUT KAM ↓ only in hip-focused group (*p* = 0.003).
Chung & Jeong, 2018 [51]	To compare CFE vs. NIE in chronic neck pain.	41 patients with chronic neck pain.	Craniocervical flexion exercise.	Neck isometric exercise.	Pain, disability, lordosis, ROM.	Assessor-blinded RCT.	CFE superior for pain, endurance, lordosis; no NDI difference.
Liu et al., 2024 [43]	To evaluate home-based RT + aerobic exercise in sarcopenia.	86 older adults with sarcopenia.	Home-based RT + walking (12 weeks).	Usual activity.	Strength, 6MWD, function.	Parallel RCT.	Significant improvements in strength and 6MWD; no body composition changes.
Watson et al., 2018 [19]	To assess HiRIT on BMD in postmenopausal women.	101 women with low bone mass.	HiRIT (8 months).	Low-intensity home exercise.	LS and FN BMD.	Single-blind RCT.	HiRIT superior for BMD and function; high compliance; no fractures.
Fitzgerald et al., 2016 [17]	To assess the effects of MT and booster sessions in knee OA.	300 adults with knee OA.	Exercise ± MT ± boosters.	2 × 2 factorial comparison.	WOMAC (primary).	Multi-center factorial RCT.	No WOMAC difference at 1 year; MT and boosters improved short-term outcomes.
Tumilty et al., 2016 [15]	To assess PBM + EE in Achilles tendinopathy.	80 adults with midportion Achilles tendinopathy.	PBM + EE (varied frequency).	Placebo laser + EE.	VISA-A.	Double-blind factorial RCT.	PBM + low-frequency EE superior (*p* < 0.001); all groups exceeded MCID.
Sevier & Stegink-Jansen, 2015 [52]	To compare Astym vs. EE in lateral elbow tendinopathy.	107 adults (113 elbows).	Astym therapy.	Eccentric exercise.	DASH (primary).	Parallel-group RCT with crossover.	Astym superior for DASH and grip strength; effects sustained to 12 months.
Abbott et al., 2015 [16]	To assess the MT and booster sessions added to exercise in knee OA.	75 adults with knee OA.	Exercise ± MT ± boosters.	2 × 2 factorial comparison.	WOMAC (primary).	Factorial RCT.	MT and boosters are superior individually; combined, MT + booster is not superior.
Dejaco et al., 2017 [53]	To compare eccentric vs. conventional RC exercise.	36 adults with RC tendinopathy.	Isolated eccentric exercise.	Conventional exercise.	Constant–Murley score.	Single-blind RCT.	Both groups improved; no between-group differences.
Harding et al., 2020 [54]	To assess HiRIT vs. IAC in men with low bone mass.	93 men ≥ 45 yrs.	HiRIT.	IAC and usual activity.	Bone geometry and strength.	Semi-randomized trial.	HiRIT improved femoral neck cortical thickness; no serious adverse events.
DeVita et al., 2018 [55]	To assess the quadriceps strengthening effects on knee biomechanics.	30 adults with knee OA.	Quadriceps strengthening.	Usual activity.	Quadriceps force, WOMAC.	RCT.	Strength and symptoms improved; no biomechanical change.

Note: ↑ indicating an increase and ↓ indicating a decrease; Abbreviations: ACL, anterior cruciate ligament; AOFAS, American Orthopaedic Foot and Ankle Society score; BMC, bone marrow concentrate; BMD, bone mineral density; CLBP, chronic low back pain; CFE, craniocervical flexion exercise; DASH, Disabilities of the Arm, Shoulder and Hand questionnaire; DVJ, drop vertical jump; EE, eccentric exercise; ESWT, extracorporeal shockwave therapy; FN, femoral neck; HiRIT, high-intensity resistance and impact training; HPQ, Health and Work Performance Questionnaire; IAC, isometric axial compression; IMAT, intermuscular adipose tissue; KAM, knee abduction moment; LS, lumbar spine; MCIC, minimal clinically important change; MVIC, maximal voluntary isometric contraction; NDI, Neck Disability Index; NIE, neck isometric exercise; NMT, neuromuscular training; OA, osteoarthritis; ODI, Oswestry Disability Index; PBM, photo biomodulation; PRP, platelet-rich plasma; RC, rotator cuff; RMVIC, relative maximal voluntary isometric contraction; ROM, range of motion; RT, resistance training; SANE, Single Assessment Numeric Evaluation; SPADI, Shoulder Pain and Disability Index; SWE, shear wave elastography; VAS, visual analog scale; VISA-A, Victorian Institute of Sport Assessment–Achilles; WOMAC, Western Ontario and McMaster Universities Osteoarthritis Index.

**Table 3:** Risk of bias assessment of included studies.

Author & Year	Randomization Process	Deviations from Intended Interventions	Missing Outcome Data	Measurement of Outcome	Selection of Reported Result	Overall Risk of Bias
Heron et al., 2017 [44]	Low	Some concerns	High	Low	Low	Some concerns
Vahdatpour et al., 2018 [45]	Low	Low	Low	Low	Low	Low
Gatz et al., 2021 [14]	Low	Low	Low	Some concerns	Low	Some concerns
Seo et al., 2021 [46]	Some concerns	Low	Low	Low	Some concerns	Some concerns
Pereira et al., 2019 [47]	Low	Low	Low	Low	Low	Low
Messier et al., 2022 [48]	Low	Low	Low	Low	Low	Low
Khalid et al., 2022 [49]	Some concerns	Some concerns	Low	Some concerns	Low	Some concerns
Cui et al., 2023 [20]	Low	Low	Low	Low	Low	Low
Demir Benli et al., 2022 [13]	Low	Some concerns	Low	Some concerns	Low	Some concerns
Centeno et al., 2024 [50]	Low	Some concerns	Low	Some concerns	Low	Some concerns
Ford et al., 2025 [18]	Low	Low	Low	Low	Low	Low
Chung & Jeong, 2018 [51]	Some concerns	Low	Low	Some concerns	Low	Some concerns
Liu et al., 2024 [43]	Low	Low	Low	Low	Some concerns	Some concerns
Watson et al., 2018 [19]	Low	Low	Low	Low	Low	Low
Fitzgerald et al., 2016 [17]	Low	Low	Low	Low	Low	Low
Tumilty et al., 2016 [15]	Low	Low	Low	Low	Low	Low
Sevier & Stegink-Jansen, 2015 [52]	Low	Some concerns	Low	Some concerns	Low	Some concerns
Abbott et al., 2015 [16]	Low	Low	Low	Low	Low	Low
Dejaco et al., 2017 [53]	Low	Some concerns	Low	Some concerns	Low	Some concerns
Harding et al., 2020 [54]	High	Some concerns	Low	Low	Low	High
DeVita et al., 2018 [55]	Low	Some concerns	Low	Some concerns	Low	Some concerns

**Table 4:** Summary of pain outcomes across included studies.

Study	Condition/Population	Intervention	Comparator	Pain Outcome Measure	Main Finding
Demir Benli et al., 2022 [13]	Midportion Achilles tendinopathy	Eccentric exercise	ESWT	VAS	Both groups improved; no consistent superiority
Tumilty et al., 2016 [15]	Midportion Achilles tendinopathy	PBM + eccentric exercise	Placebo laser + eccentric exercise	VISA-A	The PBM adjunct showed greater improvement
Gatz et al., 2021 [14]	Achilles tendinopathy	Line- or point-focused ESWT + physiotherapy	Placebo ESWT + physiotherapy	VISA-A	No between-group difference
Vahdatpour et al., 2018 [45]	Chronic Achilles tendinopathy	ESWT	Sham ESWT	VAS	Greater pain reduction only at 16-week follow-up
Sevier & Stegink-Jansen, 2015 [52]	Lateral elbow tendinopathy	Astym therapy	Eccentric exercise	Pain with activity	Astym superior
Fitzgerald et al., 2016 [17]	Knee osteoarthritis	Exercise ± manual therapy/boosters	Exercise only	WOMAC pain	Short-term benefit with MT/boosters
Messier et al., 2022 [48]	Knee osteoarthritis with obesity	Diet + exercise	Attention control	WOMAC pain	Significant pain reduction
Chung & Jeong, 2018 [51]	Chronic neck pain	Craniocervical flexion exercise	Neck isometric exercise	VAS	CFE superior
Khalid et al., 2022 [49]	Post-ACL reconstruction	Neuromuscular training	Strength training	Pain scale	NMT superior

**Table 5:** Functional and disability outcomes.

Study	Condition	Intervention	Comparator	Functional Outcome	Main Finding
Heron et al., 2017 [44]	Rotator cuff tendinopathy	Open-chain vs. closed-chain vs. ROM exercise	Active comparators	SPADI	All improved; no between-group difference
Centeno et al., 2024 [50]	Rotator cuff tear (>50%)	BMC + platelet products	Exercise therapy	DASH	Injection superior
Sevier & Stegink-Jansen, 2015 [52]	Lateral elbow tendinopathy	Astym therapy	Eccentric exercise	DASH	Greater functional improvement
Abbott et al., 2015 [16]	Knee osteoarthritis	Exercise ± manual therapy/boosters	Exercise only	WOMAC	No long-term difference
Cui et al., 2023 [20]	Chronic low back pain	Digital rehabilitation	In-person physiotherapy	ODI	Equivalent outcomes
Chung & Jeong, 2018 [51]	Chronic neck pain	Craniocervical flexion exercise	Neck isometric exercise	NDI	No between-group difference
Liu et al., 2024 [43]	Sarcopenia	RT + aerobic exercise	Usual activity	Functional performance scales	Significant improvement

**Table 6:** Summary of strength, structural, biomechanical, injury-related, and work-related outcomes across included studies.

Study	Population/Condition	Intervention	Comparator	Outcome Domain	Outcome Measure	Main Finding
Seo et al., 2021 [46]	Sarcopenic older women	Resistance training	Non-exercise control	Strength/Performance	Strength, MVIC	Large improvements
Liu et al., 2024 [43]	Sarcopenic older adults	Home-based RT + walking	Usual activity	Strength/Performance	Strength, 6MWD	Significant improvement
DeVita et al., 2018 [55]	Knee osteoarthritis	Quadriceps strengthening	Usual activity	Strength/Performance	Muscle strength	Strength improved
Khalid et al., 2022 [49]	Post-ACL reconstruction	Neuromuscular training	Strength training	Strength/Performance	Strength, power	NMT superior
Watson et al., 2018 [19]	Postmenopausal women	HiRIT	Low-intensity exercise	Strength/Performance	Functional performance	HiRIT superior
Pereira et al., 2019 [47]	Office workers	Neck-specific exercise	Health promotion	Work-related performance	Productivity measures	Exercise superior
Gatz et al., 2021 [14]	Achilles tendinopathy	Point-focused ESWT	Placebo ESWT	Structural/Imaging	SWE elasticity	Elasticity increased only with point ESWT
Demir Benli et al., 2022 [13]	Achilles tendinopathy	Eccentric exercise	ESWT	Structural/Imaging	Tendon thickness, vascularity	EE maintained long-term benefit
Centeno et al., 2024 [50]	Rotator cuff tear	BMC + platelet products	Exercise therapy	Structural/Imaging	MRI healing	Greater structural healing
DeVita et al., 2018 [55]	Knee osteoarthritis	Quadriceps strengthening	Control	Biomechanical	Knee joint biomechanics	No biomechanical change
Ford et al., 2025 [18]	Female soccer athletes	NMT + biofeedback	NMT	Biomechanical/Injury risk	Knee abduction moment (KAM)	Hip-focused biofeedback effective
Watson et al., 2018 [19]	Low bone mass (women)	HiRIT	Low-intensity exercise	Structural/Bone health	Lumbar spine and femoral neck BMD	Significant gains
Harding et al., 2020 [54]	Low bone mass (men)	HiRIT	IAC/usual activity	Structural/Bone health	Femoral neck cortical thickness	HiRIT superior
Ford et al., 2025 [18]	Adolescent female soccer players	NMT + real-time biofeedback	NMT alone	Injury Risk/Prevention	Knee injury risk proxy (KAM)	Hip-focused biofeedback most effective
Pereira et al., 2019 [47]	Office workers	Ergonomics + neck exercise	Ergonomics + health promotion	Work-related outcomes	Presenteeism, productivity loss	Exercise group superior

**Table 7:** Comparative effectiveness of exercise-based interventions with and without adjunct modalities across musculoskeletal conditions: evidence from randomized controlled trials (*n* = 21).

Study (Author, Year)	Condition	Sample Size (EG/CG)	Intervention (Exercise ± Adjunct)	Outcome Measure	Pre (EG/CG)	Post (EG/CG)	Mean Difference (Between-Group)	SMD (95% CI)	Effect Magnitude	Direction of Effect
Sevier & Stegink-Jansen, 2015 [52]	Chronic lateral elbow tendinopathy (>12 weeks)	57/56 (completed: 46/44 elbows)	EG: Astym (instrument-assisted soft tissue mobilization) 2×/week for 4 weeks + supervised stretching + eccentric strengthening CG: Home-based eccentric exercise (stretching + eccentric strengthening)	DASH	29.5 ± 14.5 vs. 29.8 ± 14.6	15.4 ± 10.9 vs. 19.7 ± 11.4	5.5 (EG improvement 13.3 vs. CG 7.8; *p* = 0.047)	0.42 (0.00, 0.84)	Small–Medium	Favors EG
Tumilty et al., 2016 [15]	Achilles tendinopathy (>3 months)	80 total: 4 groups (*n* = 20 each); EG: Groups 2 & 4 (Laser); CG: Groups 1 & 3 (Placebo Laser)	Exercise: Unilateral heavy load eccentric training (Regime 1: 2×/day, 7 days/week; Regime 2: 1×/day, 2×/week) Adjunct: Photobiomodulation (Laser) vs. Placebo Laser (2×/week for 4 weeks)	VISA-A	EG (Group 4): 57.5 (95% CI: 53.3, 61.7) CG (Group 1): 56.7 (95% CI: 52.5, 60.9)	EG (Group 4): 99.0 (95% CI: 94.4, 103.5) CG (Group 1): 80.4 (95% CI: 75.2, 85.7)	18.5 (Group 4 vs. Group 1 at 12 weeks); 95% CI: [9.1, 27.9]	Cohen’s d: 2.5	Very Large	Strongly favors EG (Laser + reduced-frequency exercise)
Abbott et al., 2015 [16]	Knee osteoarthritis (OA)	Ex: 19; ExB: 19; Ex + MT: 18; ExB + MT: 19 (Total *N* = 75)	All groups: 12 sessions of multimodal supervised exercise Ex: 12 consecutive sessions (control) ExB: 8 sessions + 4 booster sessions (5, 8, 11 months) Ex + MT: 12 exercise + 12 manual therapy sessions ExB + MT: Exercise + manual therapy + boosters	WOMAC Total (0–240)	Ex: 70.9 ± 45.1 ExB: 108.4 ± 54.8 Ex + MT: 71.1 ± 42.8 ExB + MT: 93.5 ± 50.1	Mean change (1 year): Ex: 5.0 (95% CI: −14.2, 24.3) ExB: −51.1 (95% CI: −82.2, −20.0) Ex + MT: −34.2 (95% CI: −57.5, −11.0) ExB + MT: −3.3 (95% CI: −30.9, 24.2)	vs. Ex: ExB: −46.0 (95% CI: −80.0, −12.0) Ex + MT: −37.5 (95% CI: −69.7, −5.5) ExB + MT: −1.5 (95% CI: −35.3, 32.3)	Not reported	Moderate (ExB, Ex + MT); No effect (ExB + MT)	Favors ExB and Ex + MT; No additional benefit for combined
Dejaco et al., 2017 [53]	Rotator cuff tendinopathy	20/16 (post: 19/15)	EG: Isolated eccentric training (2 exercises: supine external rotation with elastic band + “empty-can” abduction) twice daily + stretching CG: Conventional exercise (6–8 exercises including scapular stabilization + concentric strengthening) once daily + stretching	Constant Murley (CM); VAS	CM: 72.5 ± 17.7 vs. 78.9 ± 8.5 VAS: 39.0 ± 18.5 vs. 42.0 ± 27.0	CM: 86.9 ± 16.8 vs. 88.8 ± 8.1 VAS: 19.1 ± 24.5 vs. 19.8 ± 18.5	CM: 4.6 (95% CI: −1.9 to 11.0) VAS: 2.4 (95% CI: −15.2 to 19.9)	CM: −0.14 [−0.82, 0.54] VAS: −0.03 [−0.71, 0.65]	Trivial/No effect	Neutral (no superiority; both improved)
Chung & Jeong, 2018 [51]	Chronic neck pain (≥3 months)	22/19	EG: Craniocervical flexion exercise (CFE) 30 min/day, 3×/week, 8 weeks CG: Neck isometric exercise (NIE), same duration Adjunct (both): stretching (3 × 30 s)	VAS; NDI; Lordosis (ARA); Muscle Endurance; ACROM	VAS: 4.85 ± 1.56 vs. 5.26 ± 0.99 NDI: 17.23 ± 7.54 vs. 20.11 ± 5.53 Lordosis: 18.30° ± 6.47 vs. 17.95° ± 6.04 Endurance: 9.20 ± 4.43 vs. 9.46 ± 4.00 ACROM: 103.89° ± 18.26 vs. 108.47° ± 15.00	VAS: 2.72 ± 1.28 vs. 3.97 ± 0.87 NDI: 9.05 ± 5.09 vs. 14.21 ± 5.09 Lordosis: 18.93° ± 6.45 vs. 18.19° ± 6.22 Endurance: 53.85 ± 29.22 vs. 36.95 ± 7.96 ACROM: 120.75° ± 18.97 vs. 119.01° ± 18.50	*p*-values: VAS (*p* = 0.031); Endurance (*p* = 0.003); Lordosis interaction (*p* = 0.016)	VAS: −1.13 [−1.79, −0.47] NDI: −1.01 [−1.66, −0.36] Endurance: 0.76 [0.12, 1.40] Lordosis: 0.12 [−0.50, 0.74]	Large (VAS, NDI); Moderate–Large (Endurance); Small (Lordosis)	Favors EG (CFE)
DeVita et al., 2018 [55]	Tibio-femoral knee osteoarthritis (OA)	15/15	12-week supervised quadriceps strengthening (3×/week; leg extension, leg press, forward lunge; 60–85% 3RM); CG: no intervention	Isokinetic Strength; WOMAC Pain & Function; Max Quad Force; Knee Compressive Force	Strength: 0.96 (0.39) vs. 1.12 (0.35) Pain: 6.43 (3.18) vs. 6.67 (3.31) Function: 17.7 (9.7) vs. 22.9 (10.7) Quad Force: 17.4 (5.4) vs. 21.7 (5.9) Knee Force: 34.6 (6.7) vs. 40.7 (5.4)	Strength: 1.21 (0.42) vs. 1.17 (0.40) Pain: 3.64 (2.55) vs. 7.33 (4.72) Function: 6.8 (9.5) vs. 21.1 (12.9) Quad Force: 20.2 (6.0) vs. 20.9 (5.4) Knee Force: 37.5 (6.2) vs. 38.4 (6.2)	Significant: Strength (*p* = 0.037); Pain (*p* = 0.001); Function (*p* = 0.003); 25% vs. 2% strength gain Non-significant: Quad force (*p* = 0.125); Knee compressive force (*p* = 0.161)	Strength: 0.90 WOMAC: >1.00 Quad Power: 0.91 Walking Velocity: 0.98	Large (clinical + strength); Negligible (biomechanics)	Favors EG (clinical and strength); No effect on joint loading
Watson et al., 2018 [19]	Postmenopausal women with low to very low bone mass (T-score < −1.0; osteopenia/osteoporosis)	49/52	EG (HiRIT): 8 months, 2×/week, 30 min supervised high-intensity resistance and impact training (>85% 1RM; deadlift, overhead press, back squat; 5 × 5 reps) + jumping chin-ups with drop landings CG (CON): 8 months, 2×/week, 30 min unsupervised low-intensity home-based exercise (<60% 1RM; balance, mobility, walking, stretching)	Lumbar Spine (LS) BMD; Femoral Neck (FN) BMD	LS BMD: 0.823 ± 0.108 vs. 0.816 ± 0.097 FN BMD: 0.699 ± 0.086 vs. 0.682 ± 0.059	LS BMD: 0.846 ± 0.116 vs. 0.807 ± 0.098 FN BMD: 0.700 ± 0.084 vs. 0.670 ± 0.059	LS: +2.9% ± 2.8% (EG) vs. −1.2% ± 2.8% (CG) FN: +0.3% ± 2.6% (EG) vs. −1.9% ± 2.6% (CG)	Not reported LS CI: 2.1% to 3.7% (EG) vs. −1.9% to −0.4% (CG) FN CI: −0.5% to 1.0% (EG) vs. −2.7% to −1.2% (CG)	Large (bone + functional improvements; statistically significant)	Strongly favors EG (HiRIT)
Heron et al., 2017 [44]	Rotator cuff tendinopathy/shoulder impingement syndrome (SIS)	OC: 40; CC: 40; ROM: 40	Three exercise programs: Open chain resisted band exercises; Closed chain exercises; Range of movement exercises Adjunct: Capsule stretching	SPADI	ROM: 51; OC: 49; CC: 53	ROM: 42; OC: 37; CC: 44	No statistically significant difference between groups Median change: ROM: −4.0; OC: −3.5; CC: −0.5	Not reported Within-group ES: ROM: 0.49; OC: 0.56; CC: 0.63 Median change (95% CI): ROM: −4.0 [−17 to −5]; OC: −3.5 [−5 to 12]; CC: −0.5 [−3 to 15]	Moderate (within-group); No between-group effect	Neutral (all groups improved; no superiority)
Messier et al., 2022 [48]	Knee osteoarthritis (BMI ≥ 27)	414/409	Exercise (aerobic + resistance, 60 min, 3×/week) + Calorie-restricted diet (−800–1000 kcal/day) vs. Attention Control	WOMAC Pain (0–20)	7.4 ± 3.1 vs. 7.4 ± 3.1	5.0 (95% CI: 4.6–5.4) vs. 5.5 (95% CI: 5.1–5.9)	−0.6 (95% CI: −1.0 to −0.1; *p* = 0.02)	Not reported (anticipated ≈0.23)	Small (clinically modest)	Favors EG
Vahdatpour et al., 2018 [45]	Chronic Achilles tendinopathy (AT)	22/21	EG: ESWT (1500 focused + 3000 radial shockwaves) Adjunct (both groups): 4 weeks stretching + massage + eccentric training + diclofenac 100 mg/day (2 weeks) CG: Sham ESWT	VAS; AOFAS	VAS: 7.55 ± 1.76 vs. 7.70 ± 1.34 AOFAS: 64.95 ± 14.23 vs. 64.40 ± 11.96	VAS: 3.00 ± 2.15 vs. 4.30 ± 1.84 AOFAS: 85.85 ± 7.88 vs. 79.50 ± 7.53	VAS: −1.30 AOFAS: +6.35	VAS: −0.65 [−1.26, −0.04] AOFAS: 0.82 [0.20, 1.44]	Moderate–Large (VAS); Large (AOFAS)	Favors EG
Cui et al., 2023 [20]	Chronic low back pain (CLBP)	70/70	Digital Care Program vs. Conventional Physiotherapy	ODI (Median)	24.84 vs. 25.34	17.94 vs. 18.99	−0.55 (95% CI: −2.42 to 5.81)	−0.13	Small	No significant difference
Pereira et al., 2019 [47]	Office workers (neck pain/general population)	381/382	Ergonomics + Neck-specific Exercise (20 min, 3×/week, 12 weeks) vs. Ergonomics + Health Promotion	Monetised Productivity Loss; Absenteeism; Presenteeism	Loss: $1393 ± 1029 vs. $1463 ± 941 Absenteeism: 0.6 ± 1.4 vs. 0.6 ± 1.2 Presenteeism: 2.1 ± 1.3 vs. 2.3 ± 1.3	Loss: $1464 ± 1318 vs. $1563 ± 1039 Absenteeism: 0.8 ± 2.0 vs. 0.9 ± 2.0 Presenteeism: 2.0 ± 1.2 vs. 2.4 ± 1.4	Loss: −$276 (95% CI: −474 to −42) Presenteeism: −0.563 (95% CI: −0.973 to −0.154) Absenteeism (neck pain subgroup): −0.696 (95% CI: −1.237 to −0.155)	Not reported (model coefficients used)	Small–Moderate (economic + functional impact)	Favors EG
Harding et al., 2020 [54]	Osteopenia/osteoporosis in men ≥ 45 years	FN: HiRIT 34; IAC 33; CON 26 Tibia: HiRIT 32; IAC 33; CON 23	HiRIT: High-intensity resistance + impact training (deadlift, squat, overhead press, jumping chin-ups) IAC: Machine-based isometric axial compression Adjunct: Pharmacotherapy + calcium + vitamin D permitted	FN Cortical Thickness (mm); Distal Tibia BSI (g^2^/cm^4^)	FN: HiRIT 2.01 ± 0.26 vs. CON 2.09 ± 0.31 Tibia BSI: HiRIT 1.11 ± 0.24 vs. CON 1.33 ± 0.23	FN: HiRIT 2.11 ± 0.26 vs. CON 2.08 ± 0.27 Tibia BSI: HiRIT 1.10 ± 0.24 vs. CON 1.28 ± 0.23	FN: HiRIT vs. CON 5.7% (*p* = 0.028); HiRIT vs. IAC 4.9% (*p* = 0.044) Tibia: HiRIT vs. CON 4.0% (*p* < 0.001); IAC vs. CON 3.9% (*p* < 0.001) 95% CI: FN HiRIT [2.3, 8.9]; CON [−3.9, 3.7] Tibia HiRIT [−0.7, 0.9]; CON [−4.8, −2.9]	95% CI for % change: HiRIT: [2.3, 8.9] CON: [−3.9, 3.7] 95% CI for % change: HiRIT: [−0.7, 0.9] CON: [−4.8, −2.9]	Moderate–Large (site-specific; preservation vs. loss)	Favors EG (HiRIT strongest; IAC maintains vs. decline in CON)
Ford et al., 2025 [18]	ACL injury risk (KAM)	50/50	NMT + Hip Biofeedback vs. NMT	Peak KAM (CUT)	−29.5 vs. −25.0	−22.9 vs. −25.0	*p* = 0.048	−0.44	Moderate	Favors EG
Seo et al., 2021 [46]	Sarcopenia in older adult women (>65 years)	RT: 12/CG: 10	16 weeks resistance training (bodyweight + elastic band; 3x/week, 60 min/session)	Grip Strength (kg), Gait Speed (m·s^−1^), Follistatin (pg·mL^−1^), Intramuscular Fat (IMAT) (cm^2^)	Grip Strength: RT: 20.8 ± 2.93; CG: 18.6 ± 3.07 Gait Speed: RT: 0.96 ± 0.08; CG: 0.93 ± 0.09 Follistatin: RT: 2113.75 ± 409.28; CG: 2241.85 ± 669.91 IMAT: RT: 16.0 ± 4.60; CG: 16.1 ± 3.90 CG: 2241.85 ± 669.91, RT: 16.0 ± 4.60 CG: 16.1 ± 3.90	Grip Strength: RT: 24.3 ± 2.25; CG: 17.3 ± 3.61 Gait Speed: RT: 1.14 ± 0.11; CG: 0.95 ± 0.09 Follistatin: RT: 2652.85 ± 704.18; CG: 2255.45 ± 564.02 IMAT: RT: 15.4 ± 3.15; CG: 18.1 ± 3.75	Grip Strength: RT: +3.5 kg; CG: −1.3 kg Gait Speed: RT: +0.18 m/s; CG: +0.02 m/s Follistatin: RT: +539.1 pg/mL; CG: +13.6 pg/mL IMAT: RT: −0.6 cm^2^; CG: +2.0 cm^2^	Grip Strength: RT: 1.72 Gait Speed: RT: 1.84 Follistatin: RT: 0.81 IMAT: CG: 1.06	Large	Favorable (Improvement in RT; Prevention of fat increase in RT)
Liu et al., 2024 [43]	Sarcopenia	41/45	Resistance + Walking vs. Usual care	Strength; 6MWD	Strength: 16.3 (13.6–21.3) vs. 16.0 (13.8–20.9) 6MWD: 467.3 ± 55.9 vs. 412.3 ± 76.7	Strength: 18.9 (15.9–22.4) vs. 15.1 (12.3–21.1) 6MWD: 478.2 ± 50.5 vs. 417.8 ± 86.0	Strength: 1.800 (0.140–3.460) 6MWD: 57.680 (35.350–80.010)	Not reported	Significant (moderate–large inferred)	Favors EG
Centeno et al., 2024 [50]	Supraspinatus tear	34/17	BMC + PRP + PL vs. Exercise	DASH; NPS; SANE	DASH: 31.0 vs. 30.8 NPS: 3.9 vs. 4.3	DASH: 15.5 vs. 27.4 NPS: 1.8 vs. 4.5 SANE: 46.6 vs. 7.7	ΔDASH: −11.7 vs. −3.8 (*p* = 0.01) ΔNPS: −2.0 vs. +0.5 (*p* = 0.004) SANE: +50 vs. 0 (*p* < 0.001)	Not reported	Large (MCID exceeded)	Strongly favors EG
Demir Benli et al., 2022 [13]	Chronic midportion Achilles tendinopathy	32/31	Eccentric Exercise (Alfredson protocol) vs. ESWT	VAS; VISA-A; Thickness; Strain Ratio	VAS: 5.9 ± 2.4 vs. 6.3 ± 2.2 VISA-A: 52.6 ± 20.5 vs. 53.3 ± 18.4 Thickness (cm): 4.7 ± 1.2 vs. 5.9 ± 1.9 Strain Ratio: 2.1 ± 1.5 vs. 1.8 ± 1.4	VAS: 2.6 ± 2.5 vs. 2.6 ± 2.3 VISA-A: 80.0 ± 24.3 vs. 81.4 ± 15.6 Thickness (cm): 5.2 ± 2.0 vs. 5.7 ± 2.0 Strain Ratio: 3.1 ± 2.2 vs. 1.9 ± 1.7	Short-term (3 months): VAS (*p* = 0.643); VISA-A (*p* = 0.883) → NS Long-term (2 years): VAS: 1.2 ± 1.3 vs. 5.4 ± 3.5 (*p* < 0.001)	VAS: 1.193/1.276 VISA-A: 1.398/1.488 Thickness: 1.168/0.420 Strain Ratio: 0.701/0.702	Large (short-term both); Large structural effect in EG	Equal short-term; Favors EG long-term
Khalid et al., 2022 [49]	ACL reconstruction (2 months post-op)	38/38	Neuromuscular Training vs. Strength Training	NPRS; Cincinnati Score; SF-36; Hop Tests	NPRS: 6.76 ± 1.34 vs. 6.63 ± 1.28 Cincinnati: 206.05 ± 46.94 vs. 227.63 ± 56.30 SF-36: 27.5 ± 12.23 vs. 31.71 ± 13.72 Hop tests: comparable baseline	NPRS: 1.58 ± 1.45 vs. 4.21 ± 1.12 Cincinnati: 374.21 ± 28.44 vs. 346.05 ± 36.87 SF-36: 76.05 ± 10.34 vs. 68.29 ± 10.42 Hop tests: higher in EG	NPRS: −2.63 Cincinnati: +28.16 SF-36: +7.76 Hop tests: +4.56 to +19.34 6 m hop: −0.04	Not reported	Large (consistent multi-domain improvements)	Strongly favors EG
Gatz et al., 2021 [14]	Insertional or mid-portion Achilles tendinopathy	Line-focused: 24; Point-focused: 21; Placebo: 21	All groups: Daily home-based physiotherapy (eccentric + isometric exercises + static stretching) Adjunct: 4 sessions ESWT (line-focused vs. point-focused vs. placebo) over 6 weeks	VISA-A (0–100)	Line-focused: 59 (±17.73); Point-focused: 61 (±17.29); Placebo: 60 (±17.00)	Line-focused: ~77 (+18); Point-focused: ~84 (+23); Placebo: ~75 (+15)	No statistically significant difference (*p* = 0.24); F(4,116) = 1.393	Between-group: η^2^ = 0.455 Within-group: Point: η^2^ = 0.611; Line: η^2^ = 0.410; Placebo: η^2^ = 0.331	Large within-group effects; No significant between-group effect	Neutral (no superiority of adjunct ESWT)
Fitzgerald et al., 2016 [17]	Knee osteoarthritis (KOA)	Ex: 75; Ex + B: 76; MT + Ex: 75; MT + Ex + B: 74 (Total *N* = 300)	Exercise therapy (aerobic, strengthening, stretching, neuromuscular control) for all groups Adjuncts: Manual Therapy (MT) and/or Booster sessions	WOMAC Total Score (0–240; primary outcome at 1 year)	MT groups: ~85.4 (mean of 88.1 and 82.7) No MT groups: ~86.85 (mean of 87.7 and 86.0)	MT groups: ~53.5 (mean of 57.4 and 49.6) No MT groups: ~53.7 (mean of 55.4 and 52.0)	Primary (1 year): MT vs. No MT: −1.39 (95% CI: −11.11 to 8.34; *p* = 0.78) Booster vs. No Booster: −7.76 (95% CI: −17.55 to 2.03; *p* = 0.12) Short-term (9 weeks): MT vs. No MT: −9.68 (95% CI: −19.23 to −0.10; *p* = 0.048)	Not reported Secondary: OR (Responder) MT vs. No MT: 2.57 (95% CI: 1.17–5.68; *p* = 0.019) Booster vs. No Booster (1 year): OR 2.32 (reported *p* = 0.036)	Small/Non-significant (primary); Moderate short-term effect (MT); Increased responder odds	Neutral (primary outcome); Short-term benefit (MT); Booster improves responder likelihood

Note: BMD = bone mineral density; FN = femoral neck; LS = lumbar spine; BSI = bone strength index; IMAT = intramuscular adipose tissue; 6MWD = six-minute walk distance; ACROM = active cervical range of motion; ARA = absolute rotation angle; SMD = standardized mean difference; CI = confidence interval; OR = odds ratio; η^2^ = partial eta squared; MCID = minimal clinically important difference.

## Data Availability

No new data were generated or analyzed during this study. All relevant information and original contributions are fully presented within the article. Therefore, data sharing is not applicable to this manuscript.

## Supplementary Materials

The following supporting information can be downloaded at: https://www.mdpi.com/article/10.3390/life16040608/s1, PRISMA 2020 Checklist.
