# Supplementary material for: Is Exercise Enough? Evidence from Controlled Clinical Trials on Rehabilitation with and Without Adjunct Modalities for Musculoskeletal Disorders
Source: Life (Basel). 2026 Apr 7;16(4):608. doi: 10.3390/life16040608 (PMC13117354; doi:10.3390/life16040608)
Supplement: Supplementary file 1 [file life-16-00608-s001.zip › life-4198090-supplementary.pdf]

PRISMA 2020 Checklist

Section and Topic	Item #	Checklist item	Location where item is reported
**TITLE**			
Title	1	"Is Exercise Enough? Evidence From Controlled Clinical Trials on Rehabilitation with and Without Adjunct Modalities for Musculoskeletal Disorders"	Title page
**ABSTRACT**			
Abstract	2	Structured abstract provided (Background, Objective, Methods, Results, Conclusion) following PRISMA 2020 for Abstracts.	Abstract section
**INTRODUCTION**			
Rationale	3	The rationale for the review is clearly described, highlighting the burden of musculoskeletal disorders and the need to evaluate whether adjunct modalities provide additional benefit beyond exercise-based rehabilitation.	Introduction (Paragraphs 1–4)
Objectives	4	To synthesise evidence from controlled clinical trials comparing exercise-based rehabilitation delivered alone versus exercise combined with adjunct modalities across musculoskeletal conditions.	End of Introduction
**METHODS**			
Eligibility criteria	5	Inclusion and exclusion criteria were specified using the PICOS framework.<br>Population, intervention, comparator, outcomes, and study design are clearly defined.	Eligibility Criteria; Table 1
Information sources	6	Databases searched: PubMed/MEDLINE, Scopus, Web of Science, Cochrane CENTRAL.<br>Reference lists manually screened.	Information Sources and Search Strategy
Search strategy	7	Search strategy described using controlled vocabulary and keywords.<br>Boolean operators specified.	Sources and Search Strategy
Selection process	8	Two-stage screening process (title/abstract, full-text).<br>Two independent reviewers.<br>Disagreements resolved by discussion/third reviewer.<br>PRISMA flow diagram included.	Selection Process; Figure 1
Data collection process	9	Standardized data extraction form used.<br>Two independent reviewers.<br>Cross-checking and consensus resolution are described.	Data Extraction Process
Data items	10a	Outcomes defined: pain, disability, strength, performance, biomechanics, imaging/structural changes, injury risk, work-related outcomes.	Eligibility Criteria: Tables 4–6
	10b	Extracted data included study characteristics, sample size, intervention details, comparator, and follow-up duration.	Data Extraction Process; Table 2
Study risk of bias assessment	11	The Cochrane Risk of Bias 2 (RoB 2) tool was used.<br>Two independent reviewers.<br>Five bias domains assessed.	Risk of Bias Assessment; Table 3
Effect measures	12	Between-group differences, p-values, and validated clinical scales are reported. No pooled effect sizes due to heterogeneity.	Results: Tables

PRISMA 2020 Checklist

Section and Topic	Item #	Checklist item	Location where item is reported
			4–6
Synthesis methods	13a	Studies grouped by outcome domains.	Data Synthesis Methods
	13b	Narrative synthesis used due to heterogeneity.	Data Synthesis Methods
	13c	Tabular synthesis (Tables 2–6).	Data Synthesis Methods
	13d	Meta-analysis not performed due to clinical and methodological heterogeneity.	Data Synthesis Methods
	13e	Subgroup comparisons conducted narratively (e.g., tendinopathy, OA, sarcopenia).	Data Synthesis Methods
	13f	No formal sensitivity analysis performed..	Data Synthesis Methods
Reporting bias assessment	14	Formal publication bias assessment not conducted due to the absence of a meta-analysis.	Data Synthesis Methods
Certainty assessment	15	No GRADE analysis performed due to heterogeneity and absence of pooled effect sizes.	Methods section
**RESULTS**			
Study selection	16a	21 controlled clinical trials were included.<br>PRISMA flow diagram provided.	Results: Figure 1
	16b	Reasons for exclusion are documented in the flow diagram.	Figure 1
Study characteristics	17	Detailed study characteristics provided.	Table 2
Risk of bias in studies	18	Domain-based risk of bias reported.	Table 3
Results of individual studies	19	Summary statistics and main findings are presented in structured tables.	Tables 4–6
Results of syntheses	20a	Characteristics and risk of bias summarized narratively.	Results section
	20b	Direction of effect described (exercise vs exercise + adjunct).	Results section
	20c	Heterogeneity was explored narratively by condition and intervention type.	Results section
	20d	No sensitivity analysis performed.	Results section
Reporting biases	21	Because no quantitative meta-analysis was conducted due to substantial heterogeneity, formal statistical assessment of publication bias (e.g., funnel plots or Egger's test) was not performed; however, potential selective reporting was evaluated using the Cochrane Risk of Bias 2 tool.	Methods section
Certainty of evidence	22	Certainty not graded using GRADE due to heterogeneity.	Discussion (Methodological Considerations)
**DISCUSSION**			

PRISMA 2020 Checklist

Section and Topic	Item #	Checklist item	Location where item is reported
Discussion	23a	General interpretation provided in the context of prior evidence.	Discussion - Main synthesis
	23b	Heterogeneity, blinding limitations, and adherence reporting issues were discussed.	Methodological Considerations
	23c	Systematic review only, absence of meta-analysis acknowledged.	Discussion
	23d	Clear clinical and research implications provided.	Clinical Implications and Future Research Directions
**OTHER INFORMATION**			
Registration and protocol	24a	PROSPERO registration provided: CRD420261309183	Protocol Registration section
	24b	Registered in PROSPERO.	Protocol Registration
	24c	Describe and explain any amendments to information provided at registration or in the protocol.	No amendments reported.
Support	25	No funding declared.	Conflict of Interest / Funding statement
Competing interests	26	Authors declared no conflicts of interest.	Conflict of Interest
Availability of data, code and other materials	27	Extracted data and materials are available upon reasonable request (if you wish, we can strengthen this statement).	End of manuscript (can add under “Data Availability”)

From: Page MJ, McKenzie JE, Bossuyt PM, Boutron I, Hoffmann TC, Mulrow CD, et al. The PRISMA 2020 statement: an updated guideline for reporting systematic reviews. BMJ 2021;372:n71. doi: 10.1136/bmj.n71. This work is licensed under CC BY 4.0. To view a copy of this license, visit https://creativecommons.org/licenses/by/4.0/
